# Successful revision surgery for very late-onset stomal obstruction following Gomez gastroplasty: a case report

**DOI:** 10.1186/s40792-021-01293-6

**Published:** 2021-09-16

**Authors:** Yudai Hojo, Yasunori Kurahashi, Toshihiko Tomita, Tsutomu Kumamoto, Tatsuro Nakamura, Yoshinori Ishida, Hisashi Shinohara

**Affiliations:** 1 Division of Upper GI, Department of Gastroenterological Surgery, Hyogo College of Medicine, 1-1 Mukogawa-cho, Nishinomiya, Hyogo 663-8501 Japan; 2 Division of Gastroenterology, Department of Internal Medicine, Hyogo College of Medicine, 1-1 Mukogawa-cho, Nishinomiya, Hyogo 663-8501 Japan

**Keywords:** Gomez gastroplasty, Stomal obstruction, Revision surgery

## Abstract

**Background:**

Gomez gastroplasty, which was developed in the 1970s as one of the gastric restrictive surgeries for severe obesity, partitions the stomach using a stapler from the lesser towards the greater curvature at the upper gastric body, leaving a small channel. This procedure is no longer performed due to poor outcomes, but surgeons can encounter late-onset complications even decades after the surgery. Here, we report a case of very late-onset stomal obstruction following Gomez gastroplasty which was successfully treated by revision surgery.

**Case presentation:**

A 58-year-old man was referred to our institution with sudden-onset nausea and vomiting. He underwent weight loss surgery in the USA in 1979, but the details of the surgery were unclear. Esophagogastroduodenoscopy demonstrated a stoma at the greater curvature of the upper gastric body, and fluoroscopy showed retention of contrast medium in the fundus and poor outflow through the stoma. Abdominal computed tomography revealed a staple line partitioning the stomach. Considering these preoperative investigation findings and the period during which the surgery was performed, the patient was diagnosed with very late-onset stomal obstruction following Gomez gastroplasty. Supporting the preoperative diagnosis, the surgical findings revealed a staple line extending from the lesser towards the greater curvature of the upper gastric body and a channel reinforced by a running seromuscular suture on the greater curvature. Moreover, gastric torsion caused by the enlarged proximal gastric pouch was found. Re-gastroplasty involving wedge resection of the original channel was performed followed by construction of a new channel. Postoperative course was uneventful, and the patient no longer had symptoms of stomal obstruction after revision surgery.

**Conclusions:**

Re-gastroplasty was safe and feasible for very late-onset stomal obstruction following Gomez gastroplasty. Accurate preoperative diagnosis based on the patient’s interview and the investigation findings was important for surgical planning. A careful follow-up is required to prevent excessive weight regain after revision surgery.

## Background

A total of 696,191 surgical and endoluminal bariatric procedures were performed worldwide in 2018 according to the survey conducted by the International Federation for Surgery of Obesity and Metabolic Disorders [1]. Sleeve gastrectomy and Roux-en-Y gastric bypass are the standard procedures at present, but different operative procedures have been developed by surgeons, since the start of bariatric surgery in the 1950s [1, 2]. Most previously developed weight loss surgeries had poor outcomes. However, surgeons can encounter late-onset complications of these procedures, even decades later. We report a case of very late-onset stomal obstruction following Gomez gastroplasty that occurred approximately 40 years after the surgery.

## Case presentation

A 58-year-old man, who underwent weight loss surgery for severe obesity in the USA in 1979, was referred to our institution with sudden-onset nausea and vomiting. He had a 20% total weight loss (body mass index [BMI] decreased from 45.2 to 36.2 kg/m^2^) after bariatric surgery, but the details of the surgery were unclear except for the information provided by the patient that a stapler was used, and iatrogenic splenectomy was performed. The patient started complaining of epigastric discomfort 20 years after surgery despite normal esophagogastroduodenoscopy (EGD) findings. His body weight gradually decreased to less than 90 kg in the preceding 5 years and was 75 kg (BMI: 22.6 kg/m^2^) at admission. There was no history of diabetes.

**Fig. 1:**
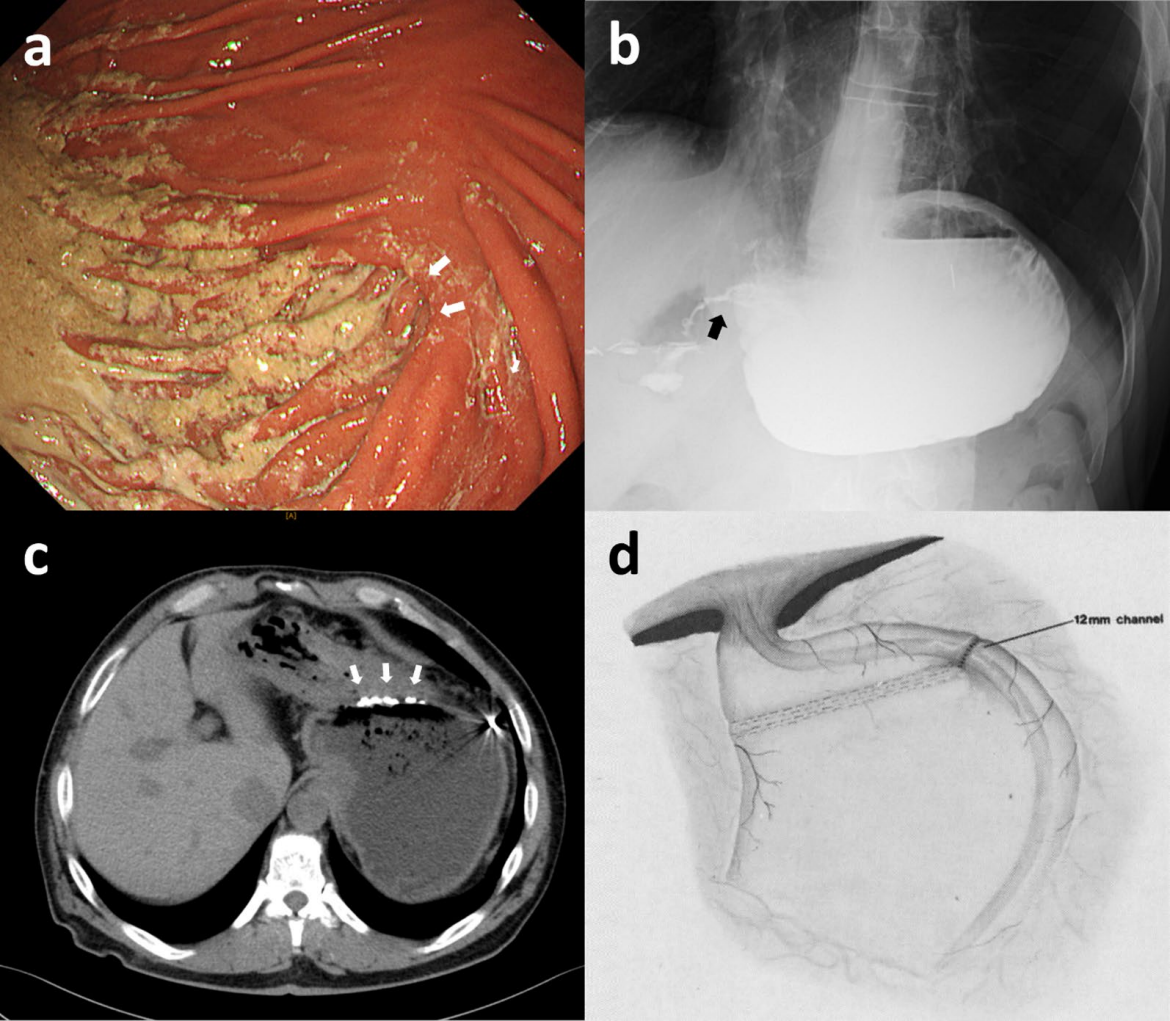
Preoperative findings. **a** Esophagogastroduodenoscopy showed a stoma (white arrows) on the greater curvature of the upper gastric body. **b** Fluoroscopy demonstrated retention of contrast medium in the fundus and poor outflow through the stoma (black arrow). **c** Abdominal computed tomography revealed a staple line (white arrows) at the upper gastric body and an enlarged fundus filled with food residue. **d** Complete image of Gomez gastroplasty (reproduced from ref. [4] with permission from Elsevier)

EGD revealed a stoma at the greater curvature of the upper gastric body (Fig. 1a), and an 8.9-mm diameter endoscope could pass through it. The stomach distal to the stoma was intact, and gastrojejunostomy was not found. Fluoroscopy demonstrated retention of contrast medium in the fundus and poor outflow through the stoma (Fig. 1b). Abdominal computed tomography revealed a staple line partitioning the stomach at the upper gastric body from the enlarged fundus filled with food residue (Fig. 1c). Considering the preoperative investigation findings and the period when the surgery was performed, the patient was diagnosed with very late-onset stomal obstruction following Gomez gastroplasty (Fig. 1d).

**Fig. 2:**
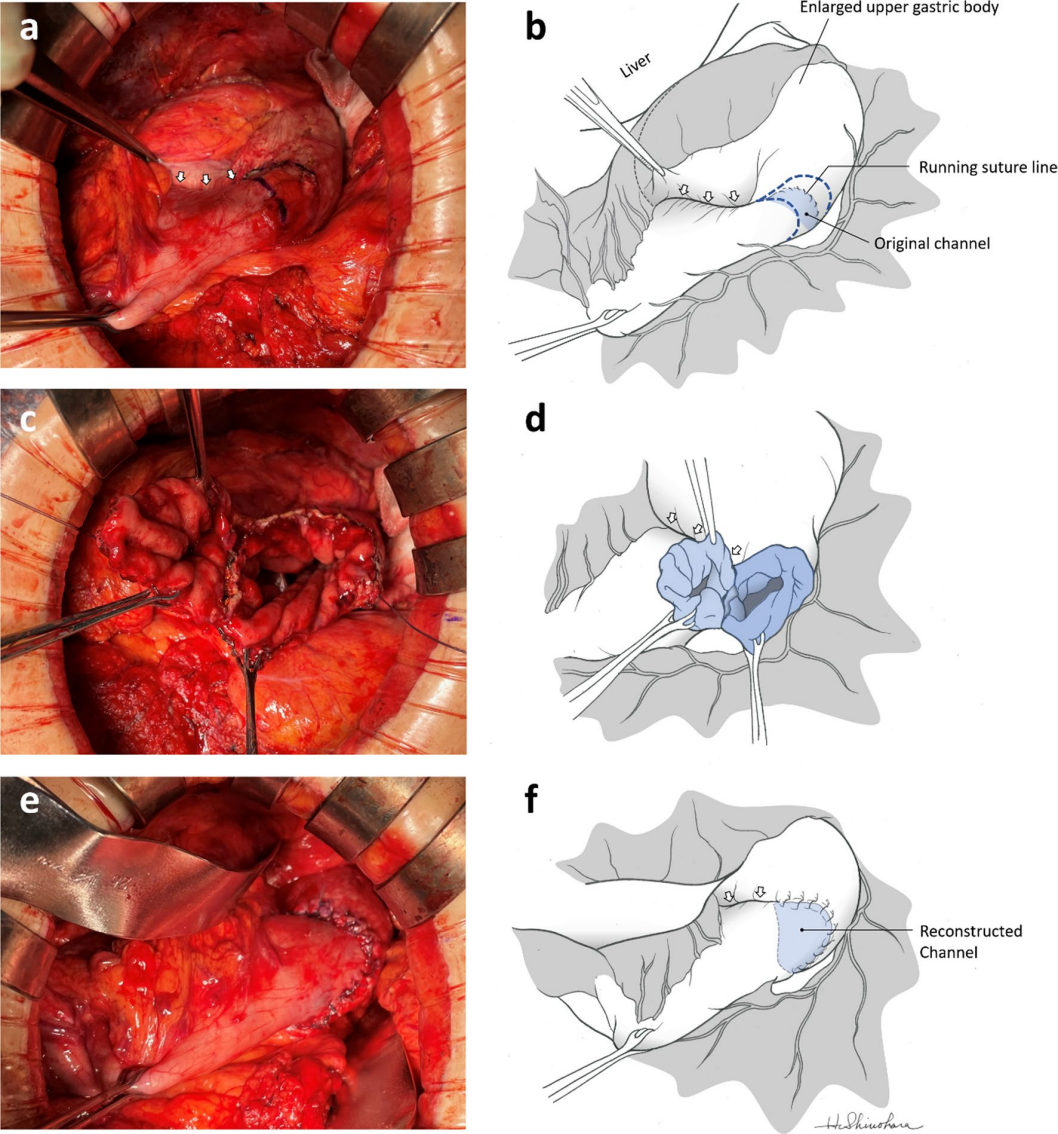
Surgical findings (**a**, **c**, **e**) and corresponding schemas (**b**, **d**, **f**). **a**, **b** Staple line, indicated by white arrows, was extending from the lesser towards the greater curvature of the upper gastric body. The channel was located on the greater curvature and reinforced with a running seromuscular suture. The resection line was designed as indicated by the broken blue lines in (**b**). **c**, **d** Original channel was resected and the diameter of the new channel increased to 30 mm. **e**, **f** New channel was constructed by double-layer anastomosis with interrupted sutures

**Fig. 3:**
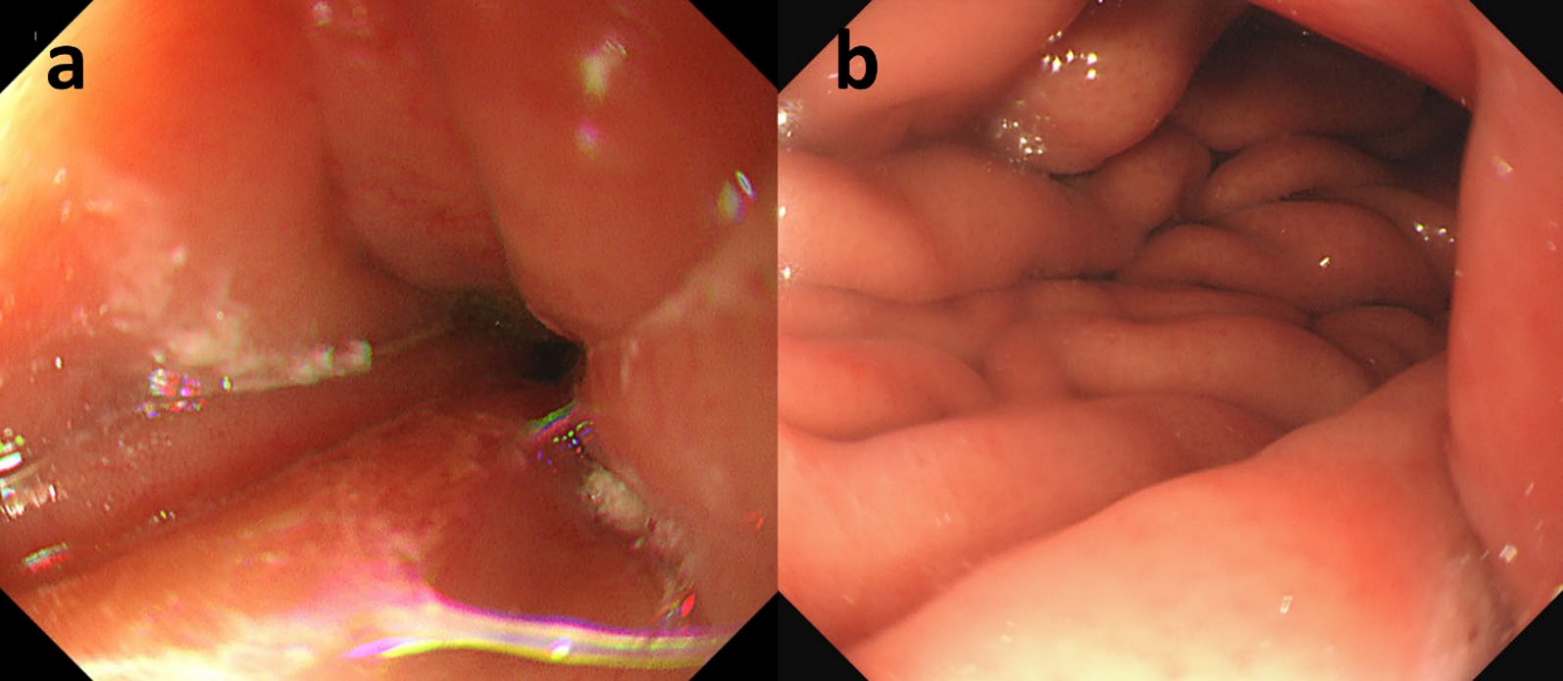
Endoscopic images of the original (**a**) and revised channels (**b**)

For revision surgery, laparotomy was performed and a midline incision from the xiphoid process to the umbilicus was performed. Severe intra-abdominal adhesions due to the initial surgery, including splenectomy, were found. The stomach had been partitioned by a staple line extending from the lesser towards the greater curvature of the upper gastric body, and a running seromuscular suture was observed around the greater curvature channel (Fig. 2a, b). Wedge resection of the original channel was performed, and the diameter of the new channel increased to 30 mm (Fig. 2c, d). The new channel was constructed by double-layer anastomosis with interrupted sutures (Fig. 2e, f). The duration of surgery was 253 min, and blood loss was 135 mL. Postoperative course was uneventful, and the patient was discharged 7 days after surgery. EGD performed at 3-month follow-up showed a well-formed new channel and no food residue in the proximal gastric pouch (Fig. 3a, b).

## Discussion

During Gomez gastroplasty, which was developed in the 1970s as one of the gastric partitioning surgeries for severe obesity, a 60 mL proximal gastric pouch and 12-mm greater curvature channel reinforced with a circumferential seromuscular suture are created [3, 4]. Stomal obstruction including channel stenosis is a common long-term complication of gastroplasty, and it occurs at a rate of 2–19% after Gomez gastroplasty in accordance with the previous reports [5, 6]. Moreover, vomiting, staple-line disruption, and dilatation of channel or proximal gastric pouch were reported as other long-term complications following Gomez gastroplasty [5]. Stomal obstruction generally occurs within 3 months to a year after the surgery [7]. This case is important, because it shows that bariatric surgeons can encounter stomal obstruction following Gomez gastroplasty, even decades after the procedure. In the present case, gastric torsion caused by the slowly enlarging proximal pouch as well as channel stenosis might have resulted in stomal obstruction, because an ordinary 8.9-mm diameter endoscope could pass through the channel in the preoperative investigation. Buckwalter et al. reported that poor blood flow around stoma by dividing short gastric arteries to mobilize the fundus might be one of the factors causing stomal obstruction [6]. Therefore, splenectomy could cause stomal obstruction by limiting the blood flow in the fundus. However, the significance of the effect of splenectomy in our case was not certain, because all reported cases with stomal obstruction were reoperated within 30 months after the primary surgery [6].

Endoscopic balloon dilatation is a less invasive treatment for stomal obstruction. However, most patients with non-stenotic stomal obstruction following Gomez gastroplasty (77.7%, 7 of 9 cases) required a re-surgery despite performing endoscopic dilatation and other conservative treatments [6]. Therefore, we chose surgery as the most effective treatment option for our case. Although conversion from gastroplasty to gastric bypass is effective for unsatisfactory weight loss or weight regain caused by stomal dilatation and/or proximal pouch enlargement [7, 8], information regarding revision surgery for stomal obstruction is scarce. We considered the following surgical options for our case: (i) re-gastroplasty; (ii) gastrojejunal bypass with Roux-en-Y with/without distal gastrectomy; and (iii) conversion to Roux-en-Y gastric bypass. Among these, we chose re-gastroplasty as a safer and more feasible option, because the intra-abdominal adhesions were too severe for performing other procedures, and the gastric wall of the proximal pouch had been damaged by progressive dilatation over decades. Considering the effect of gastric torsion, size of the channel was revised to 30 mm in diameter, which was 2.5 times larger than the original one, to prevent restenosis. Moreover, external reinforcement of the new channel was not performed for the same reason. As a result, the patient no longer had symptoms of stomal obstruction and was able to consume a normal solid diet. His weight increased from 75 to 90 kg (BMI increased from 22.6 to 27.1 kg/m^2^) in 3 months after revision surgery. He required continuous postoperative dietary counseling to prevent excessive weight regain after revision, because abnormal eating behavior may be present even decades after the initial surgery.

## Conclusions

We successfully diagnosed complications of classical bariatric surgery performed decades ago despite extremely poor preoperative information, and treated the patient with revision surgery, thereby eliminating any further difficulties with eating. A careful follow-up is needed to prevent excessive weight regain.

## Data Availability

Not applicable.

## Abbreviations

BMI: Body mass index
EGD: Esophagogastroduodenoscopy
